# Supplementary material for: Transcriptional Control of Subcutaneous Adipose Tissue by the Transcription Factor CTCF Modulates Heterogeneity in Fat Distribution in Women
Source: Cells. 2023 Dec 30;13(1):86. doi: 10.3390/cells13010086 (PMC10778492; doi:10.3390/cells13010086)
Supplement: Supplementary file 1 [file cells-13-00086-s001.zip › cells-2773793-supplementary.pdf]

**qPCR Primer**	**Sequence**
CYC1for	CGAGGTTTTGACTCTCGTGG
CYC1rev	AACACTACCCCGCGAAGC
COXC8Afor	GCCCATAGCGTAGGAAGGTG
COXC8Arev	CGCGGAAGTCTCTCGGAAAT
ACO2for	CCATTGCGTTCACAGGGTTC
ACO2rev	CGCCTTTAAAGCCTTTGGGC
PHB2for	GAAAGAAAGGGCAGCGGAAG
PHB2rev	CGAAGTTCGGGTCCGTAGTG
PRDX3for	TGGAGACCCTGAGGGAGAAA
PRDX3rev	GTTGGGGCGACAGTAGGAAA
EBF1for	AGTGATTTGCAGGCTTTCCCT
EBF1rev	TCCCTTTGGGGTTAGTGTGC
NDUFA6for	GGTGCTGGCGGTAGAAGTAG
NDUFA6rev	GGGTTGTGGAGTGGATGCTT
GAPDHfor	GAAGGTGAAGGTCGGAGTC
GAPDHrev	GAAGATGGTGATGGGATTTC

Supplemental Table S1: Primers used for ChIP-qPCR assay in Figure 4.

Supplemental Figure S1.

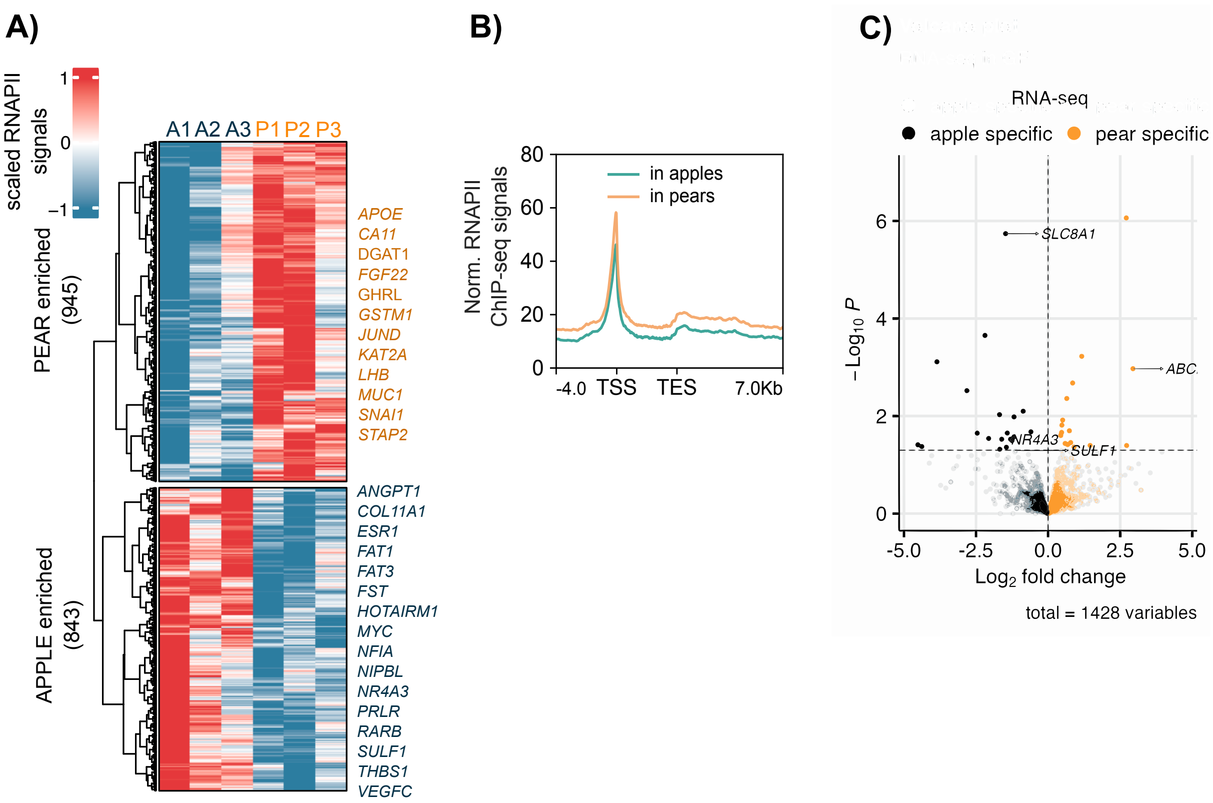

Supplemental Figure S1. Differential RNAPII binding at transcription start site (TSS) between apple- versus pear-ADSCs shows body shape-specific transcriptional signatures in gluteofemoral depot. **A)** Clustered heatmap showing normalized values of 1,788 body shape specific RNAPII bindings in each subject in GF-ADSCs. A=Apple, P=Pear. Z-score were obtained at the TSS (±2kbp). **B)** Metagene profile representing normalized RNAPII ChIP-Seq signals in apple and pear samples at genes with body shape specific RNAPII binding. TSS=Transcription Start Site. **C)** Volcano plot showing differentially expressed genes between apple and pear samples based on RNA-seq data. The genes with body shape enriched RNAPII binding are represented in colors.

Supplemental Figure S2.

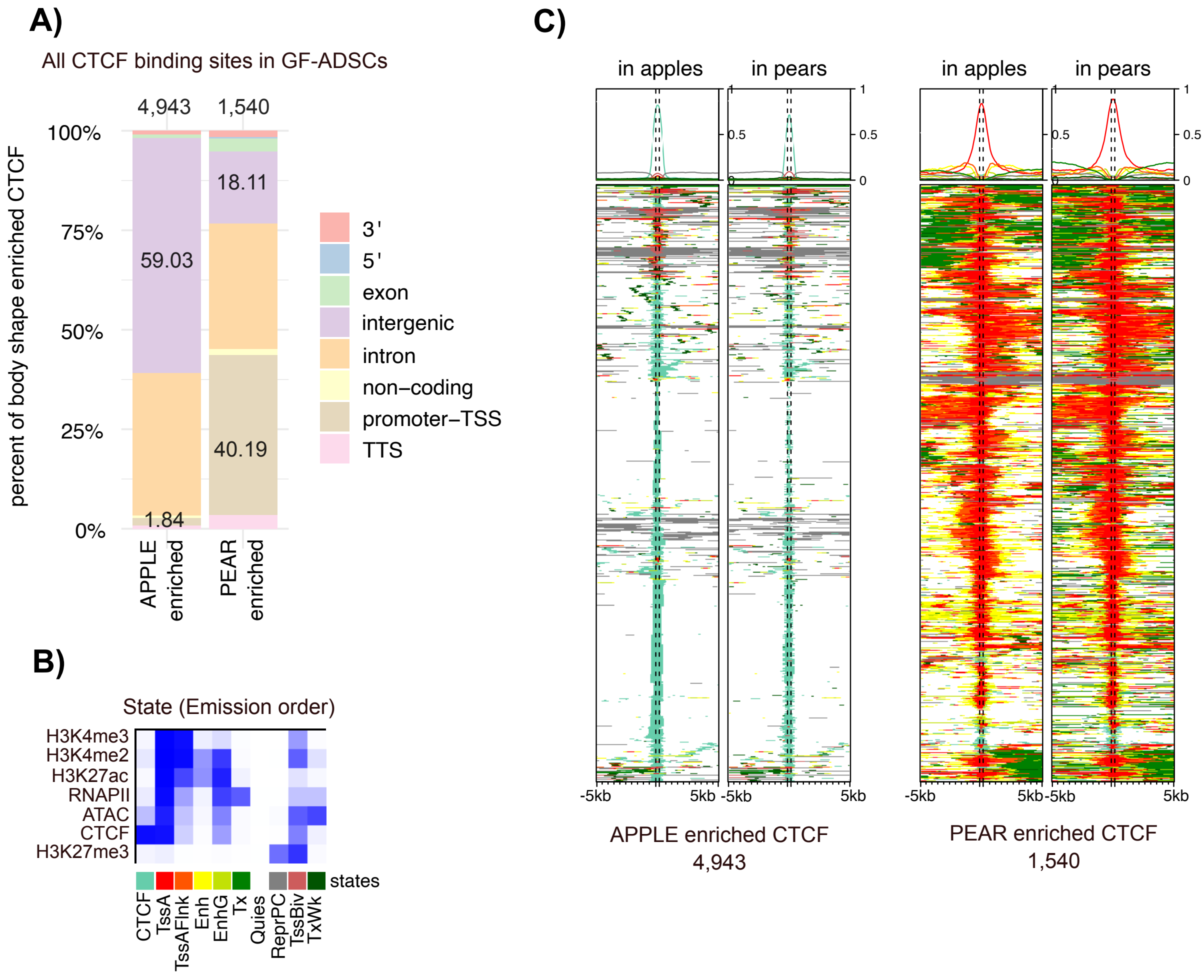

Supplemental Figure S 2. Apple and pear enriched CTCF binding sites in GF-ADSCs. **A)** Bar graph represents the genomic distribution of the body-shape enriched CTCF binding sites in GF-ADSCs. TSS=Transcription Start Site, TTS=Transcription Termination Site. **B)** Heatmap of the emission parameters in which each column corresponds to a different state, and each row corresponds to a different mark for four histone modifications (H3K4me2, H3K4me3, H3K27me3, H3K27ac), ATAC-seq, RNAPII and CTCF. **C)** ChromHMM heat map representation showing signals around the apple (left side) and pear (right side) enriched CTCF binding sites in apple and pear samples (n=6).

Supplemental Figure S3.

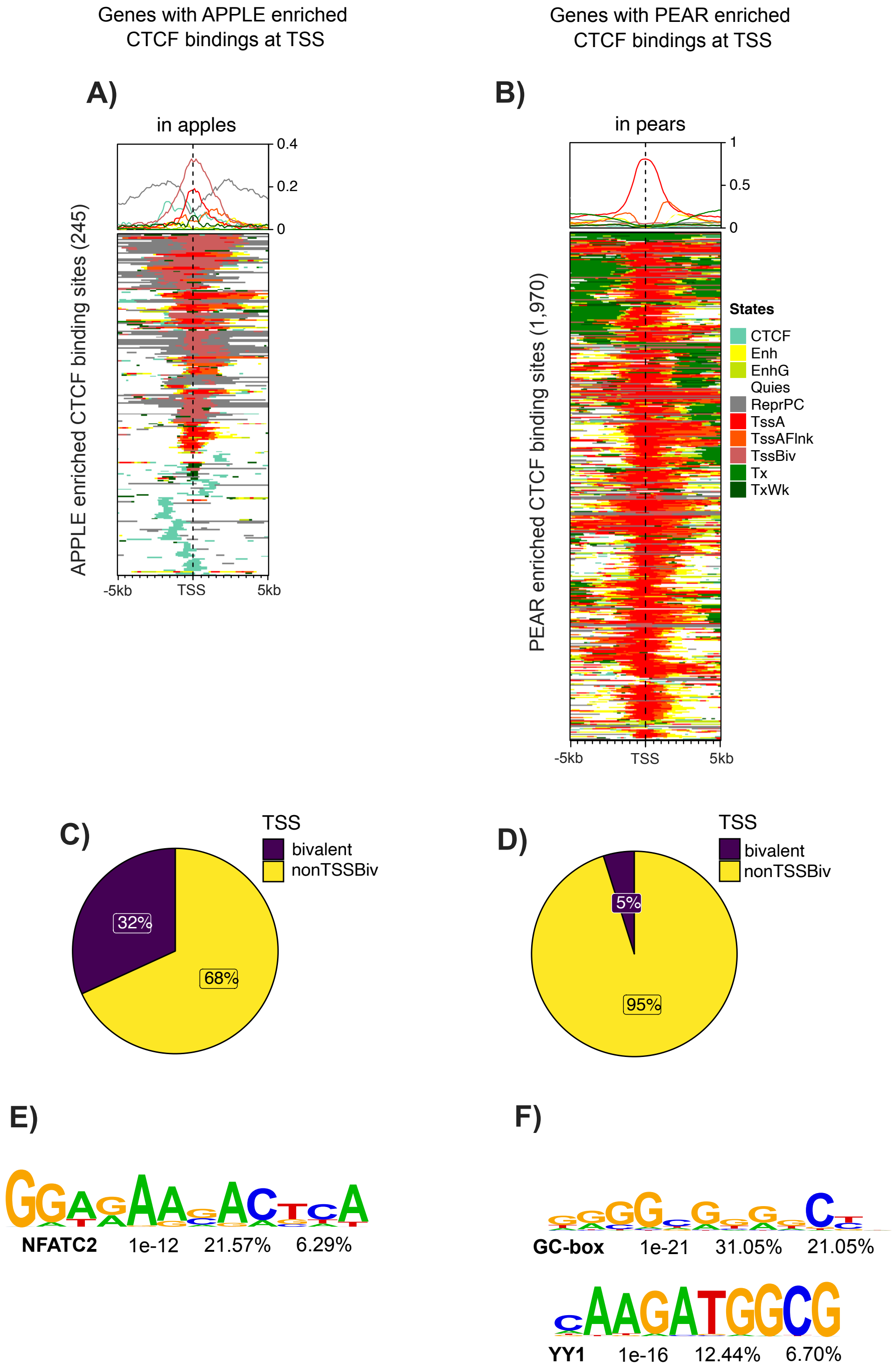

Supplemental Figure S3. Apple enriched CTCF binding is associated with bivalent TSS and pear enriched CTCF binding is associated with active TSS in gluteofemoral depot. ChromHMM heat map representation showing signals **(A)** around the 245 apple-enriched CTCF binding sites in apple samples (n=3) and **(B)** ChromHMM heat map representation showing signals around the 1,970 pear enriched CTCF binding sites in pear samples (n=3). Pie chart showing **(C)** the percentage of apple-specific CTCF binding genes with bivalent TSS and **(D)** the percentage of pear-specific CTCF binding genes with bivalent TSS. Motif enrichment analysis for the apple **(E)** and pear **(F)** enriched CTCF binding peaks. Enriched motif matrices are presented along with the p-value. The percentage of each motif found in the target and background genomic regions are indicated.

Supplemental Figure S4.

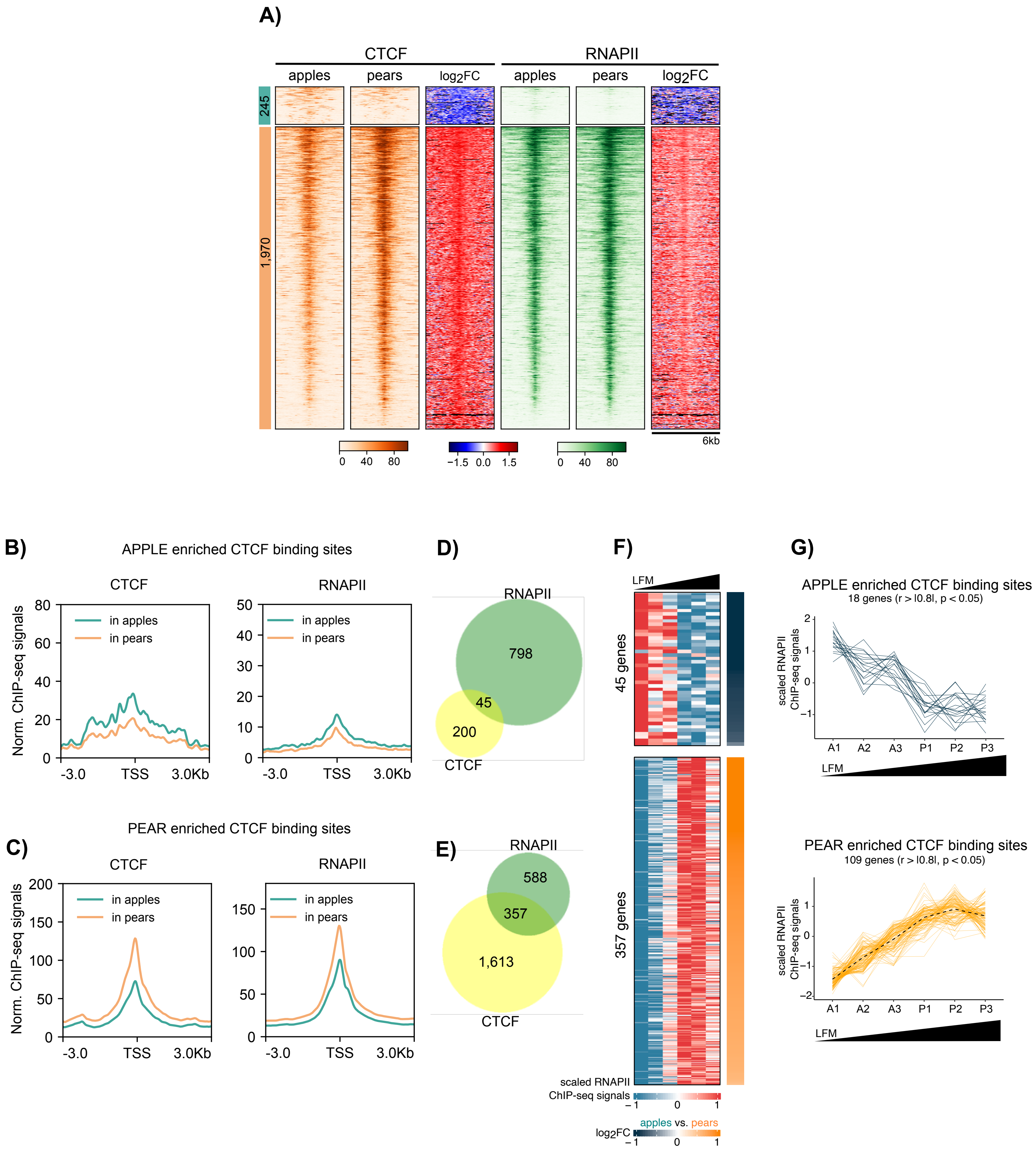

Supplemental Figure S4. Association between body shape enriched CTCF and RNAPII binding at transcription start site (TSS) in gluteofemoral depot. **A)** Heatmap showing CTCF (orange) and RNAPII (green) enrichment in apple and pear samples at apple (left heat maps) and pear (right heat maps) specific CTCF binding sites within ±3kb frame of TSS. Log2 FC heatmaps represent the ratio of CTCF or RNAPII signals between apple and pear samples. **B)** Metagene profile representing normalized CTCF and RNAPII ChIP-seq signals in apple (teal) and pear (orange) samples around genes TSS with apple enriched CTCF binding. **C)** Metagene profile representing normalized CTCF and RNAPII ChIP-seq signals in apple (teal) and pear (orange) samples around genes TSS with pear enriched CTCF binding. **D)** Proportional Venn diagrams showing number of genes with only RNAPII (green), only CTCF (yellow) or simultaneously RNAPII and CTCF (intersection) apple enriched binding sites at their TSS. **E)** Proportional Venn diagrams showing number of genes with only RNAPII (green), only CTCF (yellow) or simultaneously RNAPII and CTCF (intersection) pear enriched binding sites at their TSS. **F)** Heatmaps showing individual RNAPII ChIP-seq signal at TSS of 45 and 357 genes with respectively apple and pear CTCF/RNAPII enriched binding sites. Subjects are sorted by LFM. Log2 FC heatmaps (right side) represent the ratio of RNAPII signals between apple and pear samples; orange indicates higher signal in pear samples, blue indicates higher signal in apple samples. **G)** Lineplots showing normalized RNAPII ChIP-seq signals at gene TSS of apple (top) or pear (bottom) enriched CTCF/RNAPII binding sites in each subject ordered by LFM. N=6 subjects. Only genes presenting correlation between RNAPII and LFM are shown (Spearman, rho > |0.8|, p-value < 0.05).

Supplemental Figure S5.

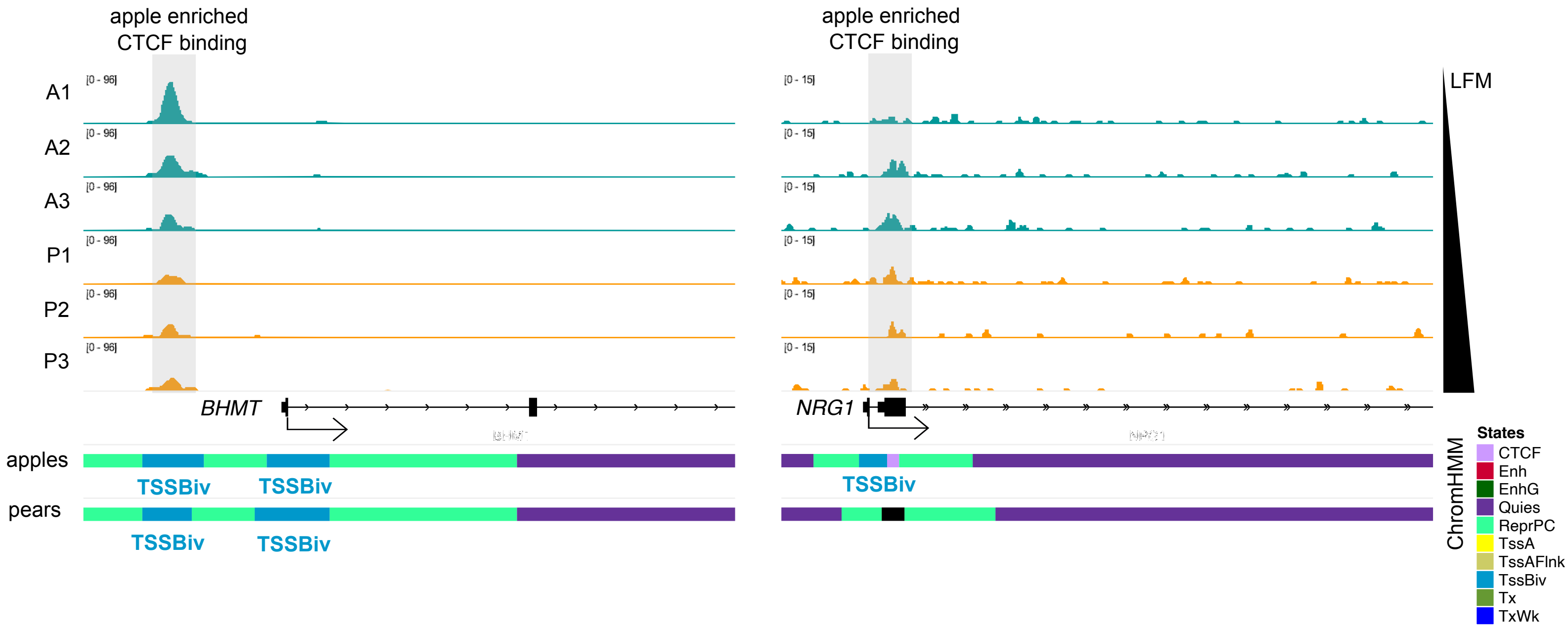

Supplemental Figure S 5. IGV screenshots showing decreased of CTCF signals at BHMT and NRG1 promoter in parallel of LFM increased. ChromHMM representation shows the presence of bivalent TSS state at their promoter. Legend for ChromHMM was the same as Fig.6D.
